# Influence of lifestyle patterns on depression among adults with diabetes: a mediation effect of dietary inflammatory index

**DOI:** 10.1186/s12889-024-19319-7

**Published:** 2024-07-03

**Authors:** Baoping Wang, Yuxin Fan, Xin Wang, Xiangru Zeng, Sha Zeng, Hongwei Jia, Yin Li, Chenlin Dai

**Affiliations:** 1 Department of Endocrinology and Metabolism, Tianjin Medical University General Hospital, No. 154, Anshan Road, Heping District, Tianjin, 300052 China; 2 Department of Endocrinology, Tianjin Dongli Hospital, Tianjin, 300300 China; 3 Department of Endocrinology and Metabolism, Tianjin Medical University General Hospital Airport Hospital, Tianjin, 300000 China; 4 Dept. Maternal, Child and Adolescent Health, School of Public Health, Tianjin Medical University, Tianjin, 300070 China

**Keywords:** Dietary inflammatory index (DII), Depression, Lifestyle patterns, Diabetes, Nhanes

## Abstract

**Background:**

Lifestyle has become a crucial modulator in the management of diabetes and is intimately linked with the development and exacerbation of comorbid depression. The study aimed to analyze lifestyle patterns and their impact on depression in individuals with diabetes and to explore the role of the Dietary Inflammatory Index (DII) in the relationship between lifestyle patterns and depression.

**Methods:**

Data was attained from the National Health and Nutrition Examination Survey (NHANES) between 2009 and 2020. A latent class analysis (LCA) was performed on 3,009 diabetic adults based on lifestyle behaviors. A generalised linear model (GLM) was employed to analyse the effects of different lifestyle patterns on depression. The mediation effect model was utilised to examine the relationship between lifestyle patterns, DII and Patient Health Questionnaire-9 (PHQ-9) scores.

**Results:**

The cohort was divided through LCA into unhealthy lifestyle (44.53%), unhealthy but non-alcohol use (48.06%) and healthy but smoking (7.41%) groups of lifestyle behaviors, the unhealthy but non-alcohol use group was identified as a risk factor for depression (OR = 1.379, 95%CI = 1.095 ~ 1.735, *P* = 0.006). The DII partially mediated the relationship between the unhealthy but non-alcohol use group and PHQ-9, and fully mediated the relationship between the healthy but smoking group and PHQ-9, with effect coefficients of − 0.018 (95%CI: −0.044 ~ − 0.001) and − 0.035 (95%CI: −0.083 ~ − 0.001).

**Conclusions:**

Lifestyle patterns significantly influence the occurrence of depression among diabetes patients. The dietary inflammation plays a varying mediating role between different lifestyle patterns and depression. Restricting pro-inflammatory diets or encouraging anti-inflammatory diets, combined with the promotion of healthy lifestyle practices, may be an effective method for preventing and alleviating symptoms of depression among patients with diabetes.

## Background

The global prevalence of diabetes is increasing. The International Diabetes Federation (IDF) estimates that 536.6 million individuals had diabetes (diagnosed or undiagnosed) in 2021, a number projected to surge by 46% to 783.2 million by 2045 [1]. Diabetes, a major unrelieved daily burden [2], is increasingly associated with various psychological findings. These can escalate into psychological syndromes specific to living with diabetes and further intensify into diagnosable psychiatric disorders [3]. Notably, the likelihood of depression in individuals with diabetes is approximately double to triple that of the general population [4, 5]. The World Health Organization’s 11th edition of the International Classification of Diseases and Related Health Problems (ICD-11) defines depression as a syndrome characterized by a range of identifiable clinical symptoms and observed behaviors associated with distress and personal functional impairment [6]. Depression has been linked to adverse clinical profiles, including poorer glycemic control, dietary habits and adherence to exercise in individuals with diabetes [7].

The etiology of comorbid depression in diabetes is multifaceted, encompassing genetic, biological, psychological and social factors [8]. Of particular interest is the role of lifestyle factors, which are critical modulators of both diabetes management and the development and exacerbation of depression [9]. Individuals with diabetes often exhibit poorer lifestyle behaviors, including reduced physical activity, disrupted sleep patterns, and increased substance use (e.g., alcohol consumption and tobacco smoking), all of which can negatively influence mental health trajectories and increase the risk of depression [10]. For instance, chronic sleep disturbances can disrupt hormonal balance and promote inflammation, both implicated in the pathogenesis of depression [11]. Similarly, smoking, a detrimental lifestyle habit, impacts nicotinic acetylcholine receptors (nAChRs) in the brain, potentially influencing pathways involved in stress response, anxiety and mood regulation [12]. Alcohol consumption can further complicate the situation by interfering with blood glucose control and contributing to depressive symptoms, creating a vicious cycle of metabolic and mood disturbances [13]. In addition, about one in two patients with diabetes remain inactive or insufficiently active, which is also associated with an increased risk of depression [14]. Regular exercise has been shown to reduce depressive symptoms by promoting neuroplasticity and reducing inflammation [15].

In fact, diabetes as a long-term chronic disease, different individuals may have different characteristics and behavioral patterns. With a predominance of elderly patients, there is often a coexistence of multiple adverse lifestyles. Previous studies have focused on analyzing the relationship between a single behavior and depression [9–14], whereas there is a large variation in the overall lifestyle pattern among persons with diabetes, and the relationship with depression is unclear. Therefore, identifying diabetes-based lifestyle patterns is important for diabetes treatment strategies and prevention of co-morbidities.

Furthermore, within the context of diabetes management, dietary habits are paramount due to their direct impact on glycaemic control and the risk of complications [16]. However, individuals with diabetes often consume pro-inflammatory diets, which can contribute to the pathogenesis of depression [17]. The Dietary Inflammatory Index (DII) serves as a valuable tool to quantify the inflammatory potential of an individual’s diet, which can be used to assess the impact of diet on health outcomes [18]. Diets high in pro-inflammatory components, such as saturated fats, trans fats, and refined sugars, are associated with higher DII scores and increased risk of depression [19]. Conversely, diets rich in anti-inflammatory nutrients such as omega-3 fatty acids, whole grains, fruits and vegetables, correspond to lower DII scores and a reduced risk of depression [20]. These dietary patterns not only promote better glycaemic control but also provide essential nutrients that can enhance mood and cognitive function. It is noteworthy that individuals who maintain a healthier lifestyle, which includes regular physical activity, adequate sleep and limited alcohol and tobacco use, are more likely to adhere to a balanced and anti-inflammatory diet. This virtuous cycle of positive lifestyle behaviors and dietary choices can contribute to better overall health outcomes [21, 22]. Therefore, the role of diabetic lifestyle patterns for depression may be influenced by dietary inflammation, while dietary inflammation may mediate the relationship between lifestyle patterns and depression.

This study aims to provide a comprehensive understanding of how lifestyle factors and dietary inflammation influence the risk of depression in diabetes patients. We analyzed the lifestyle characteristics of patients with diabetes, explored the relationship between lifestyle characteristics and depression and investigated the role of the dietary inflammation between different lifestyles and depression. We hypothesize that the dietary inflammation may differentially influence the risk of depression in individuals with diabetes depending on their lifestyle behaviors. The findings could inform the development of targeted interventions that integrate lifestyle modifications and dietary strategies to reduce the burden of depression in this population.

## Methods

### Study population

The National Health and Nutrition Examination Survey (NHANES) is a large, cross-sectional, population-based survey designed to assess the lifestyle, nutrition and health condition of the non-institutionalised civilian US population. The NHANES protocol was approved by the National Center for Health Statistics Research Ethics Review Board, and written informed consent was obtained from all participants. Data were collected via in-person interviews and physician-performed medical examinations. Further details regarding study design and data collection are available on the NCHS website [23, 24].

**Fig. 1:**
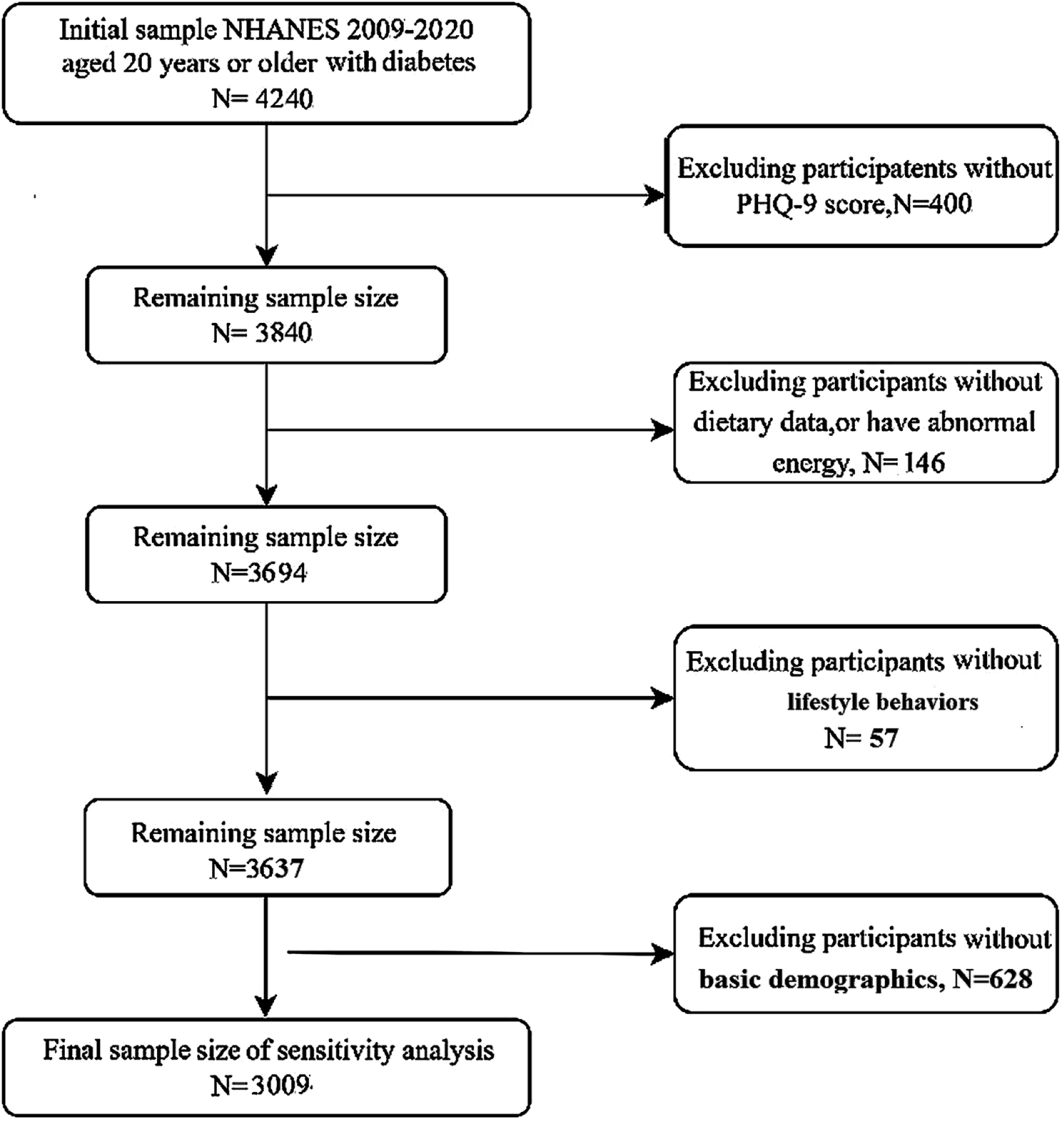
Flow chart for screening of research subjects

This study utilised NHANES data collected between 2009 and 2020. A total of 4,240 participants with physician-diagnosed diabetes were identified. Participants with missing data on lifestyle behaviors (*n* = 57), dietary intake (*n* = 146), basic demographics (*n* = 628) or outcome variables (*n* = 400) were excluded, resulting in a final analytic sample of 3,009 participants. The detailed screening process is shown in Fig. 1.

### Assessment of diabetes

The NHANES provided self-reported personal data on chronic disease conditions (adults aged ≥ 20 years), including diabetes. Participants were classified as having diabetes based on their responses to the question: “Has a doctor or other health professional ever told you that you had diabetes?” These questions were administered by trained interviewers using the Computer-Assisted Personal Interviewing (CAPI) system, which incorporates built-in consistency checks to minimise data entry errors.

### Assessment of lifestyle behavior

Data on lifestyle behaviors were collected through in-person questionnaires and 24-hour dietary recalls. Five lifestyle variables were dichotomised: cigarette smoking, alcohol drinking, sleep duration, moderate-to-vigorous physical activity (MVPA), and sedentary behavior. Smoking was regarded as unhealthy (coded as 1), which was identified by a response of “Yes” to the question, “Have you smoked at least 100 cigarettes in your entire life?” [25]. Drinking was identified by the average number of drinks on the days in which an alcoholic beverage was consumed, and an unhealthy level was defined as the consumption of more than two drinks for men and more than one drink for women (a drink = a 12 oz beer, a 5 oz glass of wine, or 1.5 oz of liquor), according to the Dietary Guidelines for Americans (coded as 1) [26]. Sleep was reported by participants using the average number of hours of sleep per day regardless of weekdays or weekends, and sleep < 6 and > 8 h was considered unhealthy (coded as 1) [27]. MVPA was self-reported and measured by the average number of minutes engaged in leisure-time moderate and vigorous activities per day. According to recommendations of the World Health Organization (WHO), < 150 min of moderate-intensity physical activity, 75 min of vigorous-intensity physical activity or an equivalent combination of moderate- and vigorous-intensity physical activity per week was defined as unhealthy (coded as 1) [28]. Sedentary behavior was assessed by the time spent sitting per day, and sitting for more than 7.5 h a day was defined as unhealthy (coded as 1) according to previous studies [28, 29].

### Assessment of dietary inflammatory index (DII)

In NHANES, dietary intake data was collected using two 24-hour dietary recall interviews. The types and amounts of foods and beverages consumed in the 24 h preceding each interview were collected, and the intakes of energy, nutrients, and other food components were then estimated from these recalls. The DII was calculated using a modified version of the method developed by Shivappa et al. [18, 30]. Briefly, this method incorporates data on 27 nutrients: alcohol, vitamins A/B6/B12/C/D/E, caffeine, carbohydrate, cholesterol, total fat, fibre, Fe, Mg, Zn, Se, MUFA, PUFA, niacin, n-3 fatty acids, n-6 fatty acids, protein, riboflavin, saturated fat, thiamin, and β-carotene. The DII scores were still available despite the nutrients applied for the calculation of DII being < 30 [18]. Individual intake of each dietary parameter was first standardised by subtracting the global mean intake (derived from a global database) and dividing by the standard deviation. These z-scores were then converted to percentiles and transformed to a symmetrical distribution ranging from − 1 to + 1. These values were doubled, subtracted from 1, and then multiplied by their respective food parameter-specific inflammatory effect score. These products were summed to obtain the overall DII score [18].

### Assessment of depressive symptoms

The Patient Health Questionnaire-9 (PHQ-9), a validated nine-item screening instrument based on the Diagnostic and Statistical Manual of Mental Disorders-IV (DSM-IV) criteria for depression, was administered in the NHANES to assess depressive symptoms over the past 2 weeks [31]. Participants rated the frequency of nine depressive symptoms experienced over the preceding two weeks using a four-point scale: “not at all” (0 points), “several days” (1 points), “more than half the days” (2 points), and “nearly every day” (3 points). Total PHQ-9 scores range from 0 to 27, with scores of 10 or higher indicative of clinically significant depressive symptoms [32].

### Covariates

The following variables were included as covariates due to their potential confounding effects: age, sex, ethnicity (non-Hispanic White/ non-Hispanic Black/ others), education level (less than high school/ high school or equivalent/ college or above), household income, and body mass index (BMI), which were obtained by self-reported. Household income, measured by the poverty income ratio (PIR), was categorised into three levels: high (> 3.5), middle (1.3–3.5), and low (≤ 1.3) [33]. BMI was categorised as follows: normal or low weight (< 25.0 kg/m^2^), overweight (25.0–29.9 kg/m^2^), or obese (≥ 30 kg/m^2^) [32].

### Data analysis

All the data were combined into one dataset according to the NHANES protocol, and data analyses accounted for the masked variance and used the recommended weighting methodology.

Latent class analysis (LCA) is a person-centered modelling method [34] that provides a more flexible, data-driven way to classify heterogeneous groups of variables according to the responses of subjects to observed variables. It not only ensures the maximization of inter-group variance and minimization of intra-group variance of classification results, but also uses objective statistical indicators to measure the accuracy and effectiveness of classification [35, 36]. Therefore, LCA is often considered a more statistically reliable clustering method that can be used to determine the optimal number of classifications for a study population [37].

LCA was deployed to identify the underlying groups based on the five lifestyle behaviors. The following criteria determine the optimal model: Akaike information criterion (AIC), Bayesian information criterion (BIC), sample-size adjusted Bayesian information criterion (aBIC), Bootstrap likelihood ratio test (BLRT), and adjusted LoMendell-Rubin likelihood ratio test (aLMR). Generalised linear model (GLM) was used to analyse the effects of different lifestyle patterns (class of lifestyle behaviors) on depression among participants with diabetes. The mediation effect model was used to analyse the relationship between lifestyle patterns, DII and PHQ-9 scores among diabetes adults. The LCA was conducted using Mplus version 8.3. The mediation effect analysis was conducted using IBM SPSS 26.0 (PROCESS Macro Model 4). All other analyses were conducted using R 4.2.2. Two-sided p-values less than 0.05 were considered to indicate statistical significance.

## Results

### Demographics

Table 1 exhibits the basic characteristics of participants with diabetes from NHANES 2009–2020 above 20 years old. Among 3,009 participants, the mean (SE) age was 61.33 (12.79) years. The mean (SE) DII was 1.16 (1.90), and 411 (13.66%) participants had depressive symptoms.

**Table 1:** Distribution of basic demographic characteristics and relevant variables of participants

Variables	Number	*N* (%)/SD
**Sex**		
Male	1600	53.17
Female	1409	46.83
**Age (year)**		61.33 ± 12.79
**Ethnicity**		
Non-Hispanic White	1080	35.89
Non-Hispanic Black	777	25.82
Other	1152	38.29
**Education Level**		
Less than high school	893	29.68
High school or equivalent	1622	53.90
College graduate or above	494	16.42
**PIR**		
Low	1074	35.69
Middle	1209	40.18
High	726	24.13
**BMI**		
Normal or low-weight	358	11.90
Overweight	845	28.08
Obesity	1806	60.02
DII		1.16 ± 1.90
**Depression**		
Yes	411	13.66
No	2598	86.34

*Note * SD, standard deviation; PIR, poverty income ratio; BMI, body mass index; DII, dietary inflammatory index

### Latent class analysis of lifestyle behavior

Table 2 displays the LCA model with 1–5 classes against the fit indicators. From the 4-class model onward, the LMR and BLRT values were insignificant, and the smallest class proportion was less than 5%. Therefore, the 3-class solution was favored due to its interpretability, and its AIC, BIC and aBIC values were the smallest, indicating a good fit.

**Table 2:** Fitting indicators for the latent class analysis

Number	AIC	BIC	aBIC	entropy	LMR_P	BLRT_P	MINOR%
1	17784.606	17814.653	17798.766	1.000			
2	17731.435	17807.538	17762.587	0.837	< 0.001	< 0.001	6.12%
3	**17704.626**	**17806.786**	**17752.770**	**0.845**	**< 0.001**	**< 0.001**	**7.41%**
4	17713.229	17851.444	17778.365	0.433	0.839	0.999	2.96%
5	17723.648	17897.920	17805.776	0.509	0.337	0.999	1.76%

*Note * AIC, Akaike Information Criterion; BIC, Bayesian Information Criterion; aBIC, Adjusted Bayesian Information Criterion; BLRT, Bootstrap Likelihood Ratio Test; aLMR, Adjusted Lo-Mendell-Rubin Likelihood Ratio Test. MINOR% is the Minimum Number of Categories percentage (%)

**Fig. 2:**
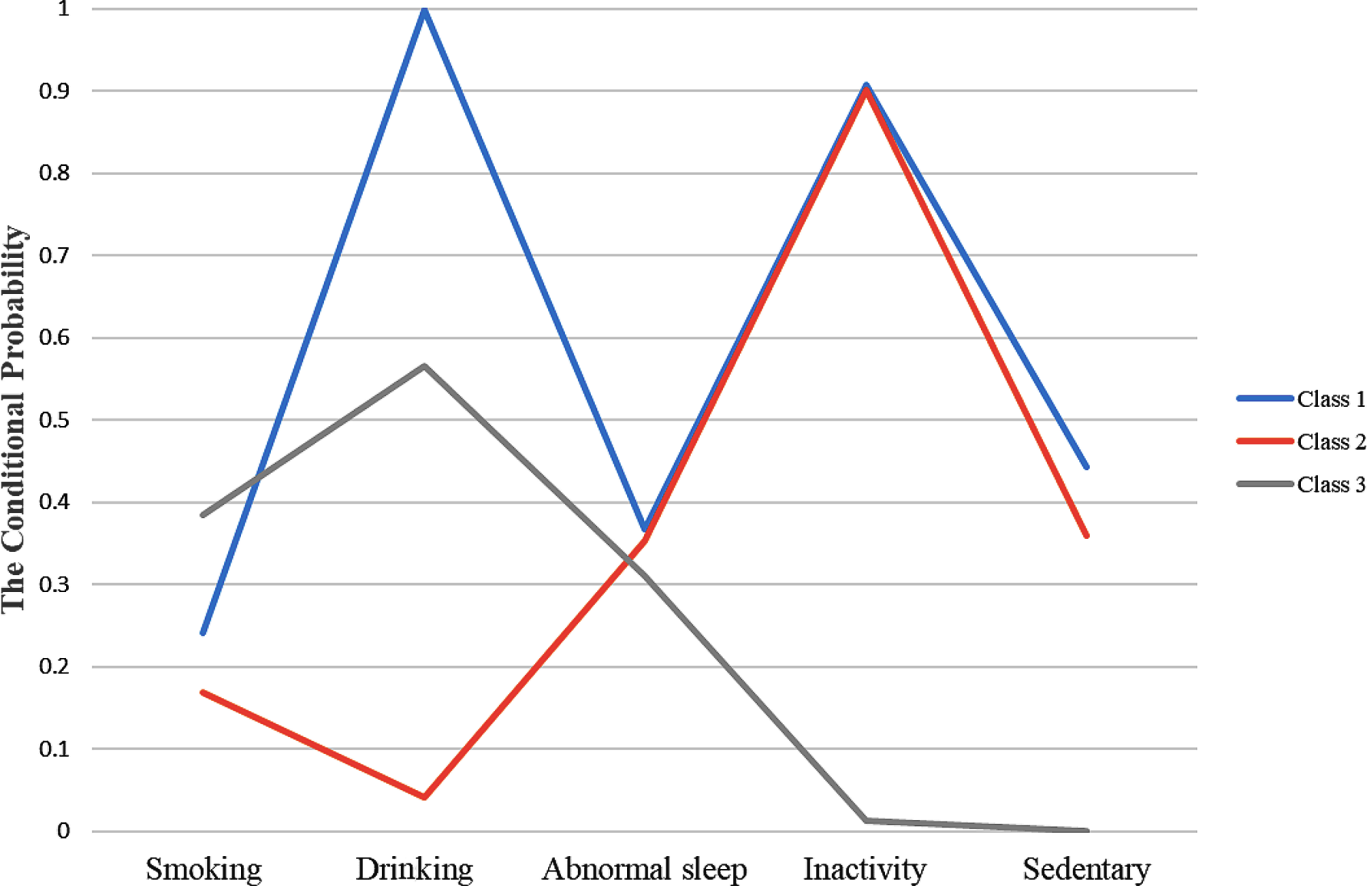
Item-response probabilities of lifestyle behaviors by the three latent class groups, United States, 2009–2020. Note: “Unhealthy lifestyle” group (Class 1) represented 44.53% of the sample (*n* = 1,340). “Unhealthy but non-alcohol use” group (Class 2) accounted for 48.06% of the full sample (*n* = 1,446). “Healthy but smoking” group (Class 3) represented 7.41% of the sample (*n* = 223)

Figure 2 shows the conditional probability of lifestyle behavior. Class 1 had high conditional probabilities for alcohol drinking (99.9%), sleep abnormalities (36.8%), inactivity (90.8%), and sedentary behavior (44.3%). Class 2 had lower conditional probabilities for alcohol drinking (4.2%) compared to Class 1. In Class 3, smoking (38.5%) had the highest conditional probability, however, sleep abnormalities (31.0%), inactivity (1.4%) and sedentary behavior (0%) had lower conditional probabilities compared to others. Therefore, Class 1 (*n* = 1,340, 44.53%) was identified as the “unhealthy lifestyle” group, Class 2 (*n* = 1,446, 48.06%) was labeled as the “unhealthy but non-alcohol use” group, Class 3 (*n* = 223, 7.41%) was defined as the “healthy but smoking” group.

### Demographic characteristics and DII among lifestyle patterns

Table 3 shows that sex (χ2 = 128.681, *P* < 0.001), age (F = 49.837, *P* < 0.001), ethnicity (χ2 = 46.485, *P* < 0.001), educational level (χ2 = 53.227, *P* < 0.001), PIR (χ2 = 14.213, *P* = 0.007) and DII (H = 14.220, *P* = 0.001) were found to be different among the three lifestyle patterns.

**Table 3:** Influence of latent class distribution on lifestyle patterns

Variables	Class 1	Class 2	Class 3	χ^2^/ F/ H	*p*-value
**Sex** ^a^				128.681	< 0.001^*^
Male	811	623	166		
Female	529	823	57		
**Age (year)** ^b^	63.18 ± 11.84	60.68 ± 13.02	54.44 ± 13.95	49.837	< 0.001^*^
**Ethnicity** ^a^				46.485	< 0.001^*^
Non-Hispanic White	563	438	79		
Non-Hispanic Black	336	389	52		
Other	441	619	92		
**Education Level** ^a^				53.227	< 0.001^*^
Less than high school	431	405	57		
High school or equivalent	731	740	151		
College graduate or above	178	301	15		
**PIR** ^a^				14.213	0.007^*^
Low	456	515	103		
Middle	544	595	70		
High	340	336	50		
**BMI** ^a^				0.827	0.935
Normal or low-weight	155	173	30		
Overweight	378	408	59		
Obesity	807	865	134		
**DII** ^c^	1.24 ± 1.86	1.15 ± 1.93	0.73 ± 1.95	14.220	0.001^*^
**Depression** ^a^				4.755	0.093
Yes	1139	1269	190		
No	201	177	33		

*Note * a: chi-square test; b: One-way ANOVA; c: Kruskal-Wallis H test; PIR, poverty income ratio; BMI, body mass index; DII, dietary inflammatory index

### Effects of lifestyle patterns and DII on depression

After adjusting for age, sex, ethnicity, educational level, PIR and DII, unhealthy but non-alcohol use (OR = 1.379, 95%CI = 1.095 ~ 1.735, *P* = 0.006) group was risk factor for depression compared to unhealthy lifestyle group (See Table 4). DII was positive for controlling depression (OR = 0.850, 95%CI = 0.801 ~ 0.902, *P* < 0.001) (See Table 5).

**Table 4:** Results of a generalized linear models of lifestyle patterns on depression

Variables	Class 2^a^			Class 3 ^a^	
	OR (95%CI)	*p*-value		OR (95%CI)	*p*-value
Depression	**1.379 (1.095, 1.735)**	**0.006** ^*^		1.011 (0.654, 1.540)	0.959

*Note * a: Reference group: Class 1; CI: confidence interval

Models adjusted for age, sex, ethnicity, educational level, PIR and DII.

**Table 5:** Results of generalized linear models of DII on depression

Variables	OR (95%CI)	*p*-value
DII	**0.850 (0.801, 0.902)**	**< 0.001** ^*^

*Note * CI: confidence interval, *Significant correlation, *P* < 0.05; DII, dietary inflammatory index

Models adjusted for age, sex, ethnicity, education level and PIR.

### Lifestyle patterns, DII and PHQ-9 scores

**Fig. 3:**
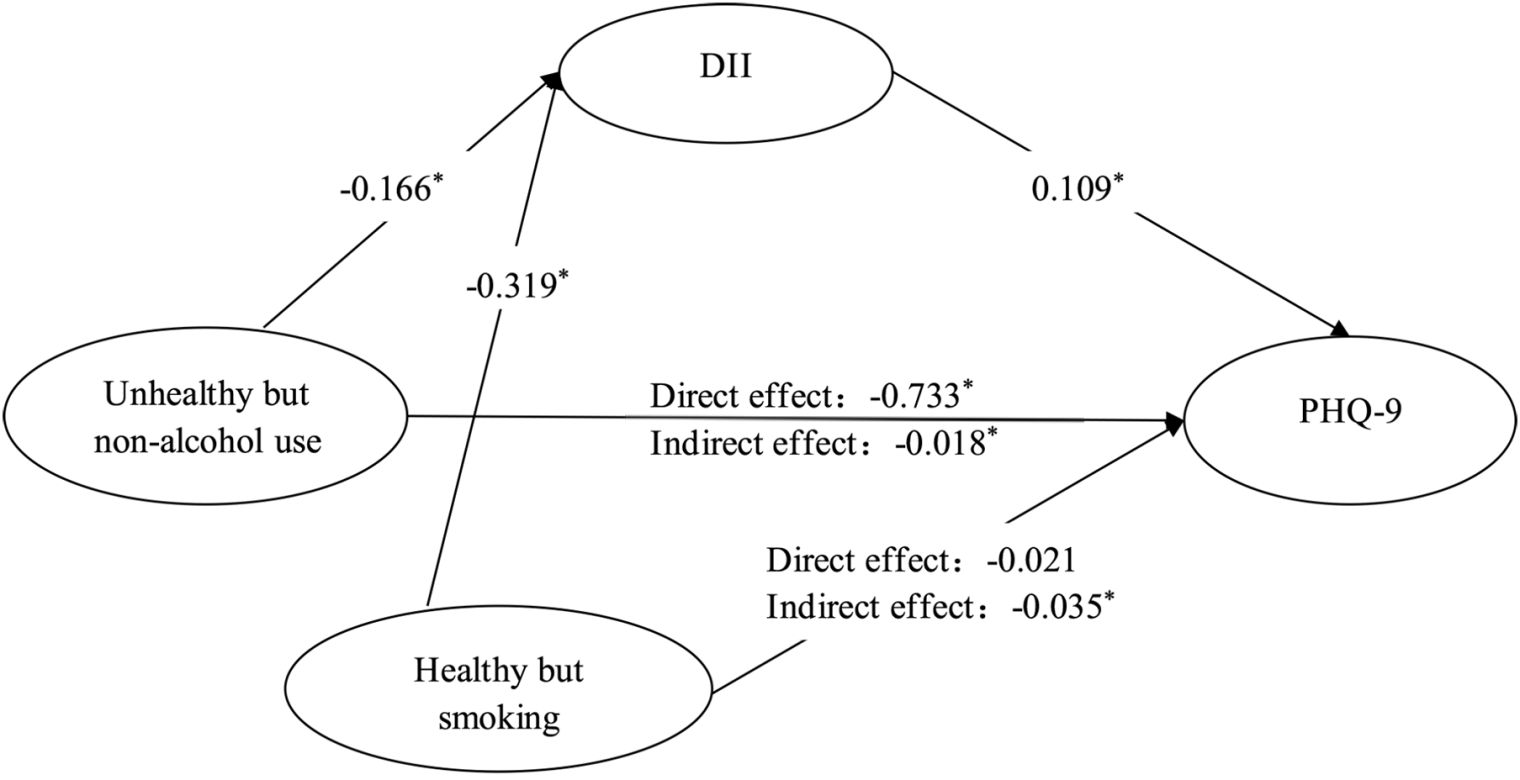
The mediating effect of DII in the relationship between lifestyle patterns and PHQ-9 among diabetes patients. *Note * *: *P* < 0.05; Reference group: Unhealthy lifestyle group

DII, lifestyle patterns and PHQ-9 were related to each other (See Table 6). Table 7 demonstrates the direct-indirect associations of lifestyle patterns, DII, and depression adjusted for demographic and socioeconomic characteristics (age, sex, ethnicity, education level, PIR and BMI). Compared to the unhealthy lifestyle group, DII partially mediated the relationship between the unhealthy but non-alcohol use group and PHQ-9, and fully mediated the relationship between the healthy but smoking group and PHQ-9, with effect coefficients of − 0.018 (95%CI: −0.044 ~ − 0.001) and − 0.035 (95%CI: −0.083 ~ − 0.001), respectively, indicating that DII explained 2.40% and 62.50% of the effect of the lifestyle patterns on PHQ-9 (See Fig. 3).

**Table 6:** Correlation matrix between variables among diabetes patients

Variables	1. Age	2. Sex	3. Ethnicity	4. Education level	5. PIR	6. BMI	7. PHQ-9	8. Lifestyle patterns	9. DII
1. Age	−								
2. Sex	−0.068^**^	−							
3. Ethnicity	−0.008	−0.013	−						
4. Education level	−0.096^**^	−0.057^**^	0.288^**^	−					
5. PIR	0.039^*^	−0.130^**^	0.115^**^	0.448^**^	−				
6. BMI	−0.205^**^	0.153^**^	−0.036^*^	0.036^*^	−0.017	−			
7. PHQ-9	−0.088^**^	0.198^**^	−0.061^**^	−0.108^**^	−0.217^**^	0.182^**^	−		
8. Lifestyle patterns	−0.151^**^	0.084^**^	−0.016	0.049^**^	0.045^*^	−0.015	−0.051^**^	−	
9. DII	0.045^*^	0.208^**^	0.030	−0.128^**^	−0.157^**^	0.032	0.093^**^	−0.053^**^	−

*Note * *Significant correlation, *P* < 0.05; **, *P* < 0.01; PIR, poverty income ratio; BMI, body mass index; DII, dietary inflammatory index

**Table 7:** The direct and indirect effects of lifestyle patterns on PHQ-9 among diabetes patients

Variables	β	Boot SE	Boot CI	
			Lower Limit	Upper Limit
**Class 2** ^a^				
Direct effect	**−0.733** ^*^	0.183	−1.091	−0.374
Indirect effect	**−0.018** ^*^	0.011	−0.044	−0.001
**Class 3** ^a^				
Direct effect	−0.021	0.348	−0.703	0.661
Indirect effect	**−0.035** ^*^	0.021	−0.083	−0.001

*Note * *: *P* < 0.05; a: Reference group: Class 1; Boot SE, bootstrap standard error; CI, Confidence Interval; Boot CI, bootstrap CI. All models were adjusted for age, sex, ethnicity, education level, PIR and BMI.

## Discussion

We analyzed the lifestyle behaviors of patients with diabetes and revealed three lifestyle patterns prevalent in this cohort of patients with diabetes, i.e. unhealthy lifestyle group, unhealthy but non-alcohol use group and healthy but smoking group. Notably, all identified lifestyle patterns included at least one unhealthy behavior. We examined the distribution of these patterns across demographic characteristics and revealed several key findings. Males were disproportionately represented in the unhealthy lifestyle group (50.09%), while females were more likely to belong to the unhealthy but non-alcohol use group (58.41%). These findings align with prior research indicating higher rates of smoking among males compared to their female counterparts [38]. Mean age differed significantly across groups, being lowest in the healthy but smoking group and highest in the unhealthy lifestyle group. This is consistent with the notion that the accumulation of unhealthy behaviors tends to increase with age, potentially contributing to the development and progression of chronic diseases [39]. As anticipated, mean DII scores were lowest in the “healthy but smoking” group and highest in the “unhealthy lifestyle” group. This supports previous observations that individuals with healthier lifestyles are more likely to adhere to anti-inflammatory dietary patterns [21, 22]. These findings underscore the interconnected nature of lifestyle behaviors and the synergistic effects they exert on health outcomes [40]. The prevalence of multiple co-occurring unhealthy behaviors within our sample highlights the need for multifaceted interventions capable of addressing these behaviors simultaneously. Such comprehensive approaches will be essential for promoting holistic health improvements and reducing the burden of comorbid chronic conditions in individuals with diabetes.

We observed significant differences in the risk of depression across lifestyle patterns. Interestingly, the unhealthy but non-alcohol use group exhibited a higher risk of depression compared to the unhealthy lifestyle group, which included individuals with unhealthy alcohol consumption patterns. This unexpected finding raises the question: why might the absence of alcohol misuse be associated with an increased risk of depression? While decades of epidemiological research have established a strong link between alcohol misuse, dependence and mood disorders, with harmful or hazardous drinking predicting more severe depressive symptoms [41], studies employing different thresholds for alcohol consumption have yielded conflicting results. Some suggest that frequent alcohol use may not necessarily increase the risk of depression [42]. Intriguingly, recent neuroimaging research indicates that alcohol consumption may influence brain function. Cheng et al. found that individuals who consume alcohol exhibit enhanced functional connectivity in brain regions implicated in cognitive control and emotional regulation, such as the medial orbitofrontal cortex and anterior cingulate cortex [43]. Conversely, individuals with depression often display disrupted connectivity in these same regions [44]. This raises the possibility that alcohol consumption, within certain limits, may exert neuroprotective effects or mitigate certain depressive symptoms. Contrary to our expectations, the healthy but smoking group did not demonstrate a protective effect against depression compared to the unhealthy lifestyle group. This suggests that additional factors, potentially operating independently or synergistically with lifestyle behaviors, contribute to depression risk in this population. Further research is warranted to elucidate the complex interplay between lifestyle factors, mediating pathways and the development of depression in individuals with diabetes.

Our findings highlight the differential role of dietary inflammation in the relationship between lifestyle patterns and depression, underscoring the need for tailored intervention strategies. We found that DII partially mediated the association between the unhealthy but non-alcohol use group and PHQ-9 scores, while fully mediating the association between the healthy but smoking group and PHQ-9 scores. These findings suggest that interventions targeting dietary inflammation may be particularly effective for reducing depression risk in individuals in the healthy but smoking group. Specifically, promoting anti-inflammatory dietary patterns and mitigating pro-inflammatory dietary habits in this subgroup could substantially reduce PHQ-9 scores and mitigate depression risk. However, individuals in the unhealthy but non-alcohol use group may benefit from a more comprehensive approach that addresses multiple unhealthy behaviors concurrently, alongside dietary modifications, to effectively reduce depressive symptoms. Chronic low-grade inflammation is a key feature of diabetes, and an anti-inflammatory diet rich in fruits, vegetables, whole grains, and omega-3 fatty acids can inhibit a variety of signaling pathways (e.g., the NF-κB and JNK pathways), leading to improved insulin sensitivity and β-cell function [45]. The significant mediating role of DII in these relationships reinforces the importance of dietary management, particularly the promotion of anti-inflammatory dietary patterns, in alleviating depressive symptoms among adults with diabetes. This aligns with a growing body of evidence supporting the mental health benefits of anti-inflammatory diets [20]. Pro-inflammatory cytokines (e.g., IL-6, TNF-α, and CRP) can cross the blood-brain barrier and affect neurotransmitter metabolism, neuroplasticity, and neuroendocrine function, leading to depressive symptoms, which can be ameliorated by an anti-inflammatory diet by decreasing pro-inflammatory cytokine levels and modulating neuroinflammation [46]. Therefore, based on our findings, it appears that an anti-inflammatory diet could be beneficial for patients with diabetes, potentially further minimizing the risk of diabetic complications when dietary inflammation is considered alongside previous conventional dietary guidelines. Future longitudinal studies are warranted to further elucidate these complex relationships and to evaluate the efficacy of targeted dietary and lifestyle interventions in individuals with diabetes. In addition, clinical trials are needed to determine the most effective strategies for translating these findings into practice, ultimately aiming to improve both mental and physical health outcomes in this population.

To our knowledge, this is the first study to investigate the interplay between lifestyle patterns, dietary inflammation and depression risk specifically among adults with diabetes. Our findings highlight the differential mediating role of DII in the relationship between lifestyle patterns and depression, underscoring the need for tailored intervention approaches. The study’s large sample size, drawn from the nationally representative NHANES database with its high-quality measurements, enhances the generalisability of our findings to the broader US adult population with diabetes. However, the present study also has several limitations. First, the DII score was calculated using 27 food parameters due to data availability constraints, potentially limiting the comprehensiveness of our dietary inflammation assessment. Second, reliance on self-reported data for both dietary intake (24-hour dietary recalls) and depression status (PHQ-9) introduces the possibility of recall bias, although this concern is somewhat mitigated by the use of validated instruments. Third, we used a cross-sectional design; thus, inferring a causal relationship would not be appropriate. Prospective studies are needed to unravel the temporal relationships between lifestyle patterns, dietary inflammation, and depression in this population. Finally, our analyses were limited by the absence of data on medication use, a potential confounder that could influence both depression and dietary behaviors.

## Conclusion

In conclusion, the current study suggests that patients with diabetes have a lifestyle pattern that includes at least one unhealthy behavior and that lifestyle patterns have implications for the development of depression. Moreover, dietary inflammation has a mediating role between different lifestyle patterns and depression with different mechanisms, emphasizing the importance of comprehensive interventions targeting different populations based on life patterns. Specifically, limiting pro-inflammatory dietary intake and promoting anti-inflammatory dietary patterns, in conjunction with broader lifestyle interventions, may be a promising approach for preventing and mitigating depression in individuals with diabetes.

## Data Availability

The publicly available data sets used in this study can be found here: https://www.cdc.gov/nchs/nhanes/index.htm.

## Abbreviations

NHANES: National Health and Nutrition Examination Survey
DII: Dietary inflammatory index
PHQ-9: Patient Health Questionnaire-9
MVPA: Moderate-to-vigorous physical activity
BMI: Body mass index
PIR: Poverty income ratio
LCA: Latent class analysis
GLM: Generalised linear model
AIC: Akaike information criterion
BIC: Bayesian information criterion
aBIC: Sample-size adjusted Bayesian information criterion
BLRT: Bootstrap likelihood ratio test
aLMR: Adjusted LoMendell–Rubin likelihood ratio test
